# Contrasting microbiomes in deep-sea scleractinian and octocoral species from the South Atlantic Ocean

**DOI:** 10.1093/sumbio/qvag048

**Published:** 2026-10-15

**Authors:** Lilian J Hill, Taissa Damasceno, Alessandro N Garritano, Flúvio Modolon, Laenne Medeiros, Caren Leite Spindola Vilela, Gustavo Duarte, Torsten Thomas, Raquel Silva Peixoto

**Affiliations:** Department of General Microbiology, Paulo de Goes Institute of Microbiology, Federal University of Rio de Janeiro, 21941-902, Rio de Janeiro, Brazil; Department of Biological Oceanography, Oceanographic Institute, University of São Paulo, 05508-120, São Paulo, Brazil; Department of General Microbiology, Paulo de Goes Institute of Microbiology, Federal University of Rio de Janeiro, 21941-902, Rio de Janeiro, Brazil; Centre de recherches insulaires et observatoire de l’environnement (CRIOBE), Université de Perpignan Via Domitia (UPVD), 66860, Perpignan, France; Centre for Marine Science and Innovation & School of Biological, Earth and Environmental Sciences, The University of New South Wales, 2033, Sydney, NSW, Australia; Department of General Microbiology, Paulo de Goes Institute of Microbiology, Federal University of Rio de Janeiro, 21941-902, Rio de Janeiro, Brazil; Department of Ecology, Environment and Geoscience, Umeå University, SE-90187, Umeå, Sweden; Department of General Microbiology, Paulo de Goes Institute of Microbiology, Federal University of Rio de Janeiro, 21941-902, Rio de Janeiro, Brazil; Department of General Microbiology, Paulo de Goes Institute of Microbiology, Federal University of Rio de Janeiro, 21941-902, Rio de Janeiro, Brazil; Department of General Microbiology, Paulo de Goes Institute of Microbiology, Federal University of Rio de Janeiro, 21941-902, Rio de Janeiro, Brazil; Red Sea Research Center (RSRC), Division of Biological and Environmental Science and Engineering (BESE), King Abdullah University of Science and Technology (KAUST), 23955-6900, Thuwal, Saudi Arabia; Centre for Marine Science and Innovation & School of Biological, Earth and Environmental Sciences, The University of New South Wales, 2033, Sydney, NSW, Australia; Department of General Microbiology, Paulo de Goes Institute of Microbiology, Federal University of Rio de Janeiro, 21941-902, Rio de Janeiro, Brazil; Red Sea Research Center (RSRC), Division of Biological and Environmental Science and Engineering (BESE), King Abdullah University of Science and Technology (KAUST), 23955-6900, Thuwal, Saudi Arabia

**Keywords:** deep-sea corals, campos basin, holobiont, host specificity

## Abstract

Deep-sea corals (DSC) are ecologically important, yet their microbiomes’ composition, structure, and host specificity remain largely unresolved in the South Atlantic, especially for octocorals. Here, we provide a comprehensive characterization of DSC microbiomes, spanning Hexacorallia (*Desmophyllum pertusum, Solenosmilia variabilis*) and Octocorallia (*Candidella imbricata*, three Malacalcyonacea species), collected at ∼710 m depth in the Campos Basin, an active oil- and gas-producing region. Abiotic characterization revealed total hydrocarbon concentrations. Seawater and sediment samples provided further environmental context. Amplicon sequencing (16S rRNA gene), combined with alpha and beta diversity analyses, differential abundance analysis, and indicator species analysis, revealed that each coral species harboured a microbial community structure distinct from the surrounding environment, with host specificity varying along a continuum. *Candidella imbricata* was the individually resolved coral species with the most specialized microbiome, dominated by *Endozoicomonadaceae*, the lowest Shannon diversity, the highest intragroup homogeneity, and the largest number of indicator amplicon sequencing variants (ASVs). *Desmophyllum pertusum* showed no exclusive indicators, overlapping broadly with environmental communities, but selectively enriched the potentially hydrocarbon-degrading family *Halieaceae*. To our knowledge, these findings establish the first microbial baseline for DSC holobionts in this region, documenting contrasting host-specificity across scleractinian and octocoral taxa.

**Sustainability statement**

This study directly contributes to UN Sustainable Development Goal 14 (Life Below Water) by establishing a comprehensive baseline information on the prokaryotic microbiomes associated with deep-sea octocorals and scleractinian corals in the Campos Basin, one of Brazil’s most active oil- and gas-producing regions. By characterizing the microbiomes of six coral species and revealing species-specific community assembly strategies, this work provides a foundation for monitoring future shifts in microbial assemblages that may signal ecosystem disturbance or resilience. By providing both characterization of prokaryotic communities and explicit documentation of the environmental hydrocarbon context in which they exist, this study enables evidence-based stewardship of deep-sea ecosystems and establishes quantifiable microbial baselines against which future monitoring programs can assess changes, directly supporting sustainable management of deep-sea energy production activities.

## Introduction

Deep-sea corals (DSCs) are mostly found at depths between 200 and 2000 m, where they play a crucial role by creating habitat for other organisms and participating in nutrient cycling and carbon sequestration (Roberts *et al*. 2006, Cathalot *et al*. 2015). These invertebrates are globally distributed and host a diverse array of microorganisms that play crucial roles in maintaining the health and ecological balance of their coral hosts (Schöttner *et al*. 2012, Middelburg *et al*. 2015, Kellogg *et al*. 2016, Ricci *et al*. 2024).

Unlike most shallow-water corals, which rely on photosynthetic dinoflagellate symbionts (Symbiodiniaceae) to supply fixed carbon and to assist in the recycling of metabolic nitrogen within the holobiont, DSCs lack this dependence on photosymbionts. Consequently, interactions with associated prokaryotes may play particularly important roles in complementing host nutrition and metabolism in the nutrient-limited deep sea. For example, chemoautotrophic prokaryotes can contribute fixed carbon to their DSC holobionts (Middelburg *et al*. 2015, Osman *et al*. 2023), while diazotrophic bacteria convert dinitrogen into bioavailable ammonia (Van Bleijswijk *et al*. 2015, Maier *et al*. 2023). Sulfur-oxidizing bacteria (e.g. *Ca. Thioglobaceae*,) further contribute to holobiont homeostasis by detoxifying hydrogen sulfide and sustaining sulfur cycling (Middelburg *et al*. 2015, Kellogg *et al*. 2016, Kellogg 2019, Osman *et al*. 2023, Vohsen *et al*. 2025).

Despite growing evidence that microorganisms contribute to key metabolic and ecological functions in DSC holobionts, the composition and structure of these microbiomes have been characterized in relatively few host taxa and ocean basins, and the degree to which they are shaped by host identity, or the surrounding environment remains unresolved. In *Desmophyllum pertusum*, the most extensively studied DSC host, the microbiome has been described as comprising a small, conserved core alongside a variable, environmentally responsive fraction (Kellogg 2019), and manipulative transplant experiments have shown that local environmental conditions can reshape this variable component within weeks (Chapron *et al*. 2020). Fewer studies have addressed octocoral hosts, but work on the North Atlantic gorgonian *Paramuricea placomus* has similarly reported host-associated bacterial communities distinct from the surrounding water column (Kellogg *et al*. 2016), and broader surveys across both stony and soft DSC taxa have identified a small set of rare, phylogenetically conserved bacteria shared across hosts despite pronounced inter-host variability in overall community composition (Kellogg 2019).

This knowledge gap is partly attributable to the logistical challenges of deep-sea sampling and is particularly pronounced in the South Atlantic Ocean (SAO). The DSCs of the SAO, particularly along the Brazilian coast, have been attracting increasing attention due to their ecological significance in forming habitats for a wide variety of marine species, as well as their vulnerability to environmental changes (Carvalho *et al*. 2023). Recently, the first characterization of deep-water scleractinian coral microbiomes from the Southwestern Atlantic was reported for four reef-building species (*D. pertusum, Solenosmilia variabilis, Madrepora oculata*, and *Enallopsamia rostrata*) across three Brazilian sedimentary basins, with *D. pertusum* showing the highest compositional stability among the species examined (Zanotti *et al*. 2024). More broadly, the microbiome of *D. pertusum* has been characterized in other ocean basins as comprising a small, conserved core alongside a variable, environmentally responsive fraction (Kellogg 2019). However, octocoral species, which are ecologically significant components of DSC communities in the region, remain entirely unstudied in this geographical context.

Filling this gap is particularly urgent in the Campos Basin, where extensive DSC habitats coexist with natural oil and gas reserves, together with exploration and production (Carvalho *et al*. 2023, Zanotti *et al*. 2024, ANP 2026). Characterizing coral-associated microbiomes together with the surrounding environmental microbial communities and local abiotic conditions is therefore important both for understanding host-microbiome specificity and for establishing baselines against which future environmental change can be assessed. Here, we used a remotely operated vehicle (ROV) equipped with purpose-built sampling tools to characterize, for the first time to our knowledge, the microbiomes of different DSC species from the Campos Basin, SAO, and assess the degree to which these communities are shaped by host identity vs. the surrounding environment.

## Methods

### Collection of coral, water, and sediment samples

This study was conducted with samples collected during a 10-day deep-sea oceanographic expedition (ProBioDeep), from August to September 2021, in the Campos Basin, Rio de Janeiro, Brazil (Fig. 1). The Campos Basin, an essential oil- and gas-producing region, is also remarkable for its DSC banks (Sumida *et al*. 2020, Carvalho *et al*. 2023).

**Figure 1:**
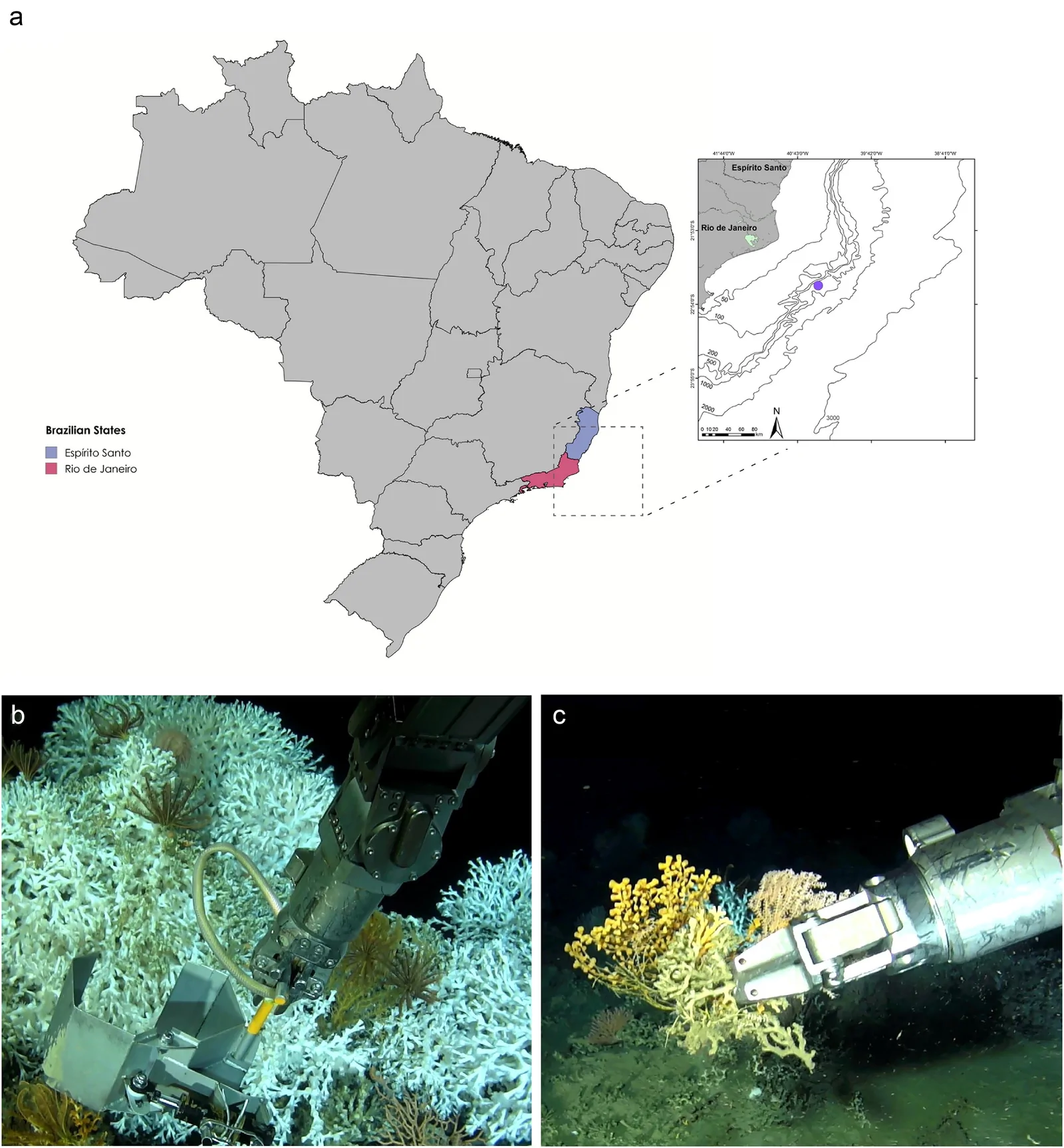
Study site and coral collection in the Campos Basin, South Atlantic Ocean. (a) Map of Brazil showing the location of the Campos Basin, between the states of Rio de Janeiro and Espírito Santo (highlighted in pink and blue, respectively). The inset shows a detailed bathymetric map of the collection area, with depth contours ranging from 100 to 3000 m. The dot indicates the location of the coral collection site (∼710 m depth). (b–c) Representative photographs taken by the ROV during collection, showing the ROV manipulator arm collecting DSC specimens from the seafloor.

The ProBioDeep expedition was carried out with the DP2 research vessel *Fugro Aquarius*, fitted with two FCV 3000 working-class ROVs capable of diving to depths of up to 3000 meters. Each ROV featured 5-axis manipulators and was customized by our team to include modifications for sample transportation, referred to as ‘BioDrawers’. These BioDrawers were 20 mm-thick high-density polyethylene compartments, designed to offer thermal insulation and divided into 12 sections. This configuration optimized the simultaneous collection of samples while preventing cross-contamination.

Using this equipment, we sampled 36 coral specimens for microbiome analysis. After surfacing, coral specimens were immediately cut with sterile equipment and rinsed with Ca- and Mg-free sterile seawater to remove loosely associated microorganisms. Coral fragments were then added to sterile cryotubes with dimethyl sulfoxide, ethylenediamine tetraacetic acid, saturated salt (DESS) solution (Yoder *et al*. 2006), and snap-frozen and stored in liquid nitrogen.

Thirteen water samples were collected with GO-FLO bottles (General Oceanics, Inc., Miami, FL, USA) ~5 m above the benthos (i.e. above the coral colonies) to minimize sediment contamination and immediately filtered through 0.22 µm filters after surfacing. The filters were placed into sterile cryotubes with DESS buffer and snap-frozen in liquid nitrogen. Sediment samples were collected with box-cores in adjacent areas to the coral sampling sites. For this study, we used eight samples from the surficial stratum (0–5 cm depth), which were stored in cryovials and snap-frozen in liquid nitrogen.

### Abiotic characterization

Water temperature and depth were recorded *in situ* using a CTD (conductivity–temperature–depth) sensor deployed during ROV dives. Water samples were collected during ROV dives and transferred to containers for chemical analysis. Amber glass containers were used for hydrocarbon samples. Nitrite, nitrate, and phosphate were determined colourimetrically using a Femto 600 Plus spectrophotometer following Aminot & Chaussepied (Aminot and Chaussepied 1983). Ammonium was measured fluorometrically using a Cary Eclipse spectrofluorometer (Varian) following Holmes *et al*. (1999). Dissolved organic carbon (DOC) was quantified by catalytic oxidation using a TOC-L elemental carbon analyser (Shimadzu Corporation, Kyoto, Japan) following Shimadzu Application Note No. 063 (Shimma and Sagawa 2019). Detection and quantification limits for each analyte are provided in Table S2. Total hydrocarbons were extracted with n-hexane following EPA SW-846 Method 8015D and quantified by gas chromatography with flame ionization detection (GC-FID; Agilent 5977B, Santa Clara, CA, USA). Aliphatic hydrocarbon fractions (C16–C21 and C21–C34) were quantified within the same analysis. Abiotic data are reported as mean ± standard deviation and are summarized in Table S2.

### DNA extraction, 16S rRNA gene sequencing and processing

DNA from each sample was extracted using the DNeasy PowerSoil Pro Kit (Qiagen, Hilden, Germany) according to the manufacturer’s instructions. The V4 hypervariable region of the 16S rRNA gene was amplified using the modified primer pairs 515F (Parada *et al*. 2016) and 806R (Apprill *et al*. 2015) and sequenced on a MiSeq platform (Illumina, San Diego, CA, USA) at the Ramaciotti Centre for Genomics (University of New South Wales, Sydney, Australia). Raw reads were deposited at the National Centre for Biotechnology Information (NCBI) under BioProject ID PRJNA1193098. Raw sequences were quality-filtered using Trimmomatic (version 0.38) after removing the first 10 bp of each read, using a sliding window of 4 bp with a minimum average quality score of 20, and reads shorter than 100 bp were discarded (Bolger *et al*. 2014, Wemheuer and Wemheuer 2017). Paired-end reads were merged using USEARCH (version 11.0.667) (22) with a minimum 8 bp overlap, and merged reads shorter than 210 bp or longer than 310 bp were removed. Merged reads with more than one ambiguous base or an expected error above 1 were discarded. The UNOISE3 algorithm (Edgar 2016) was used to cluster, denoise and generate the amplicon sequence variants (ASVs). Chimeras were removed using UCHIME3 and the SILVA version 138 database (high confidence mode) (Edgar 2016). Taxonomy was assigned to ASV sequences using the BLCA algorithm (Gao *et al*. 2017) (identity and coverage intervals set to 95%–100%) against a filtered version of the Genome Taxonomy Database (GTDB version 207, accessed on Feb/2023), in which all sequences shorter than 1000 bp were removed (Parks *et al*. 2020). Data were normalized for read counts using the DESeq2 package (Love *et al*. 2014). All non-bacterial and non-archaeal ASV sequences were removed, and reads were mapped to the remaining ASV sequences to estimate relative abundances. Data were visualized using R (version 4.4.3). Sequencing depth ranged from 22 654 to 130 916 raw read pairs per sample (3 932 891 in total). After quality filtering, chimera removal and removal of chloroplast and mitochondrial sequences, 2490 to 50 461 reads were retained per sample (median 22 579; 1 147 653 in total; Table S5). Depth was consistent among seawater (13 578 to 45 967 reads) and sediment samples (25 680 to 40 735 reads) but varied considerably among coral samples (2490 to 50 461 reads), mainly because of lower read merging success in scleractinian coral libraries.

### Molecular identification of coral specimens

Different gene regions from the total DNA extracted from coral samples were amplified based on the coral group to identify the coral specimens. For scleractinian corals, the mitochondrial cytochrome c oxidase subunit I (COI) gene was amplified using the primers and conditions previously described (Folmer *et al*. 1994) and optimized (Soto *et al*. 2021). For octocorals, a combination of the cytochrome oxidase I region (cox1) with an adjacent intergenic region (igr1) and the octocoral-specific mismatch recognition protein gene (*mtMutS*) was targeted for PCR (Polymerase Chain Reaction) amplification, following a previously published protocol (McFadden *et al*. 2011, Radice *et al*. 2016). Details about primer sequences and PCR conditions are provided in Table S3. Sequences were manually checked for quality. Voucher sequences for relevant coral genera and species were retrieved from NCBI and aligned using MUSCLE (version 3.8) (Edgar 2004). Corals were taxonomically classified based on phylogenetic trees built with suitable reference sequences using MEGA (version 12.1) (Stecher *et al*. 2026) with 1000 bootstraps (Figs S1–S3). The best-fit substitution model was selected by MEGA using the ‘Find Best DNA/Protein Models (ML)’ function prior to tree inference. Taxonomy was derived by monophyly of sample sequences with reference sequences.

### Alpha and beta diversity of microbial communities

The alpha diversity of microbial communities associated with the different coral species was measured using the Shannon diversity index and ASV richness (observed number of ASVs per sample). To account for differences in sequencing depth among samples, both metrics were calculated on rarefied data: each sample was subsampled without replacement to 2490 reads (the size of the smallest library) 1000 times using the rrarefy function, and values were averaged across iterations for each sample. Both metrics were compared across groups using the Kruskal–Wallis test followed by pairwise Wilcoxon rank sum tests with Bonferroni correction. Microbial community structure was compared between sample types using PERMANOVA (Permutational Multivariate Analysis of Variance) based on Bray–Curtis dissimilarity (999 permutations). Intragroup dispersion was assessed using the PERMDISP test. All statistical tests were performed using the vegan package in R (version 4.4.3) (Oksanen *et al*. 2016).

Bray–Curtis dissimilarity was selected as the primary metric to enable direct comparison with previous DSC microbiome studies (Kellogg *et al*. 2016, Kellogg 2019, Zanotti *et al*. 2024). Robustness of the main findings to metric choice was additionally assessed using Aitchison distance (Euclidean distance on centred log-ratio-transformed abundances following Bayesian-multiplicative zero replacement) (Palarea-Albaladejo and Martín-Fernández 2015, Fuschi *et al*. 2025).

### Differential abundance analysis

To identify prokaryotic families selectively enriched in coral holobionts relative to the surrounding environment, we calculated the log2 fold change (log2FC) of mean relative abundance for each family in each coral species compared to the surrounding seawater and sediment separately. Families were classified as coral-enriched (log2FC > 1), environment-enriched (log2FC < −1), or similar (|log2FC| ≤ 1). A pseudocount of 10^−6^ was applied to avoid zero-division errors. Additionally, pairwise DESeq2-based differential abundance analyses (Love *et al*. 2014) were performed between coral species to identify ASVs significantly enriched in each coral species relative to the others. Size factors were estimated using the poscounts method to account for zero-inflation, and model fitting was performed using a local dispersion fit. ASVs from these pairwise comparisons were considered enriched at adjusted *P* < 0.05 and |log2FC| > 1.

### Indicator species analysis

To identify ASVs significantly associated with specific coral hosts, an Indicator Species Analysis was performed using the multipatt function from the indicspecies package in R (De Caceres *et al*. 2016), with the IndVal.g metric and 999 permutations. ASVs with *P* ≤ 0.05 were considered significant and are reported in Table S1.

## Results

### Abiotic characterization of the collection site

Abiotic parameters were measured at the collection site in the Campos Basin (Table S2). Water temperature was 5.31°C ± 0.76°C at a mean depth of 710 ± 80 m. Nutrient concentrations included ammonium (1.46 ± 0.84 μmol/l), nitrite (0.06 ± 0.03 μmol/l), nitrate (27.81 ± 2.62 μmol/l), and phosphate (1.51 ± 2.03 μmol/l). DOC was 3.46 ± 3.10 μmol/l. Notably, total hydrocarbon concentrations reached 24.12 ± 9.89 μg/l, with aliphatic fractions of 5.77 ± 0.84 μg/l (C16–C21) and 17.53 ± 9.48 μg/l (C21–C34).

### Molecular identification of coral specimens

A total of 36 coral specimens were collected, molecularly identified, and assigned to six species: two Hexacorallia—*D. pertusum* (*n* = 13) and *S. variabilis* (*n* = 11)—and four Octocorallia—*Candidella imbricata* (*n* = 7), *Trachythela rudis* (*n* = 2), *Paramuricea macrospina* (*n* = 2), and *Muriceides hirta* (*n* = 1). Representative photographs of each species collected *in situ* are provided in Fig. S1. All six species were confirmed with high confidence (i.e. 100% identity) (Figs S2–S4). Given the low number of replicates of *T. rudis, P. macrospina*, and *M. hirta*, these three octocorals were grouped at the order level as Malacalcyonacea (*n* = 5) for all subsequent analyses.

### Coral microbiomes differ in composition and alpha diversity from the surrounding environment

Taxonomic analysis of Microbial community composition at the family level shows differences between coral and environmental samples (Fig. 2a). For instance, *C. imbricata*’s microbiome is dominated by *Endozoicomonadaceae* (average 74.4% ± 29%, varying from 12% to 96.7%) (Fig. S5), while this family has a much lower abundance in other hosts or water and sediments. *Desmophyllum pertusum*, in contrast, is dominated by *Halieaceae* (average 41.6% ± 28.4%). Water and sediment samples were dominated by *Pelagibacteraceae* and *Nitrosopumilaceae*, respectively.

**Figure 2:**
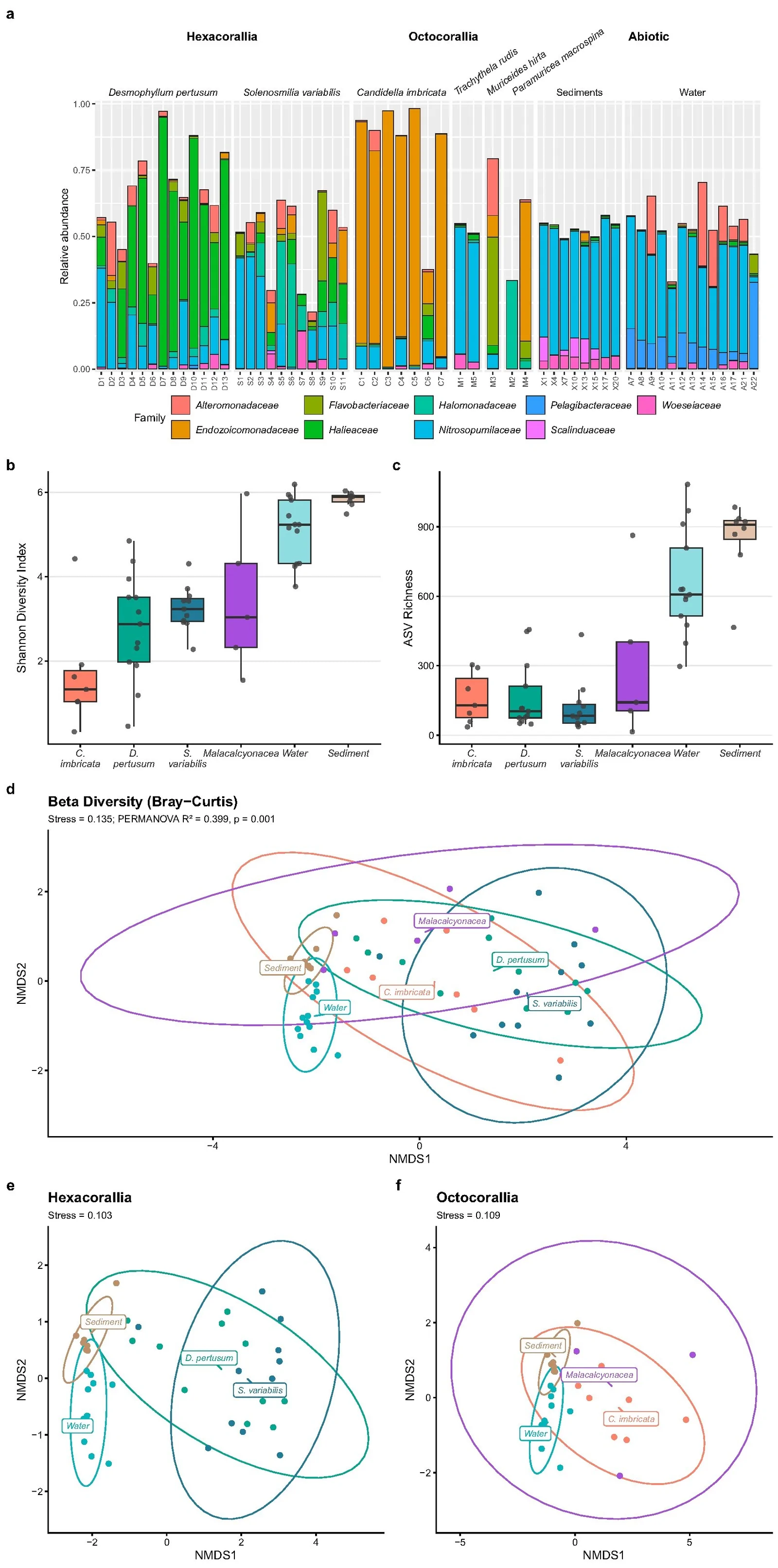
Microbial diversity and community composition of DSCs and surrounding environments from the Campos Basin. (a) Stacked bar plot showing the relative abundance of the nine most abundant bacterial families across all samples, grouped by host species (Hexacorallia and Octocorallia) and abiotic samples (Sediment and Water). Each bar represents one sample. (b) Shannon diversity index and (c) ASV richness across coral species and environmental samples, calculated on data rarefied to 2490 reads (mean of 1000 iterations). Boxes represent the interquartile range, horizontal lines the median, and points individual samples (Shannon diversity’s Kruskal–Wallis: χ² = 36.30, *df* = 5, *P* < 0.001; pairwise Wilcoxon, Bonferroni-corrected *P* ≤ 0.022 for *D. pertusum, S. variabilis*, and *C. imbricata* vs. water and sediment). (d) Non-metric multidimensional scaling (NMDS) ordination based on Bray–Curtis dissimilarity across all sample groups. Ellipses represent 95% confidence intervals around group centroids. Coral-associated microbiomes are separated from abiotic samples (PERMANOVA: *R*² = 0.399, *P* < 0.001). (e–f) NMDS ordinations restricted to Hexacorallia (e; stress = 0.103) and Octocorallia (f; stress = 0.109), respectively, and their associated abiotic samples, showing community structure within each coral order.

Quantitative diversity analyses revealed significant differences in both alpha and beta diversity of microbiomes between coral species and between corals and their surrounding environment. Coral microbiomes generally showed lower alpha diversity (Shannon index, rarefied data) than the surrounding water and sediment (Kruskal–Wallis: χ² = 36.30, *df* = 5, *P* < 0.001; Fig. 2b). Shannon diversity was significantly lower in *D. pertusum, S. variabilis*, and *C. imbricata* than in both water and sediment (pairwise Wilcoxon, Bonferroni-corrected *P* ≤ 0.022), whereas Malacalcyonacea did not differ significantly from either environmental group. Among coral species, no significant differences were detected after correction, although *C. imbricata* showed the lowest median Shannon diversity (1.33, IQR = 0.73) compared to *D. pertusum* (2.88, IQR = 1.53), *S. variabilis* (3.23, IQR = 0.55), and Malacalcyonacea (3.04, IQR = 1.99). ASV richness followed the same pattern (Kruskal–Wallis: χ² = 36.53, *df* = 5, *P* < 0.001), with environmental samples showing significantly higher richness than all coral groups except Malacalcyonacea (Fig. 2c).

### Beta diversity reveals host-specific community structure

Microbial community structure differed significantly across coral species and environmental samples based on Bray–Curtis dissimilarity, using a PERMANOVA model with sample type (i.e. each coral taxon, seawater, and sediment; six levels) as the explanatory factor (*R*² = 0.399, *P* < 0.001). Pairwise comparisons showed that microbial communities differed significantly between most coral taxon pairs and between each coral taxon and both environmental sample types, except for *C. imbricata* and Malacalcyonacea, which did not differ significantly from one another (*R*^2^ = 0.294, *P*_adj = 0.165; Table S4). Overall, separation between coral-associated and environmental communities tended to be more pronounced (*R*² = 0.376–0.515) than separation among coral species themselves (*R*² = 0.103–0.332), consistent with the ordination pattern observed by NMDS (Fig. 2d). This effect of host identity was robust to the choice of dissimilarity metric: an equivalent analysis using the compositionally-aware Aitchison distance yielded a comparable result (*R*² = 0.371, *P* = 0.001; Table S4).

Pairwise comparisons revealed that *C. imbricata* harboured the most distinct microbial community based on Bray–Curtis dissimilarity, differing significantly from *D. pertusum* (*R*² = 0.332, *P*_adj_ = 0.015), *S. variabilis* (*R*² = 0.268, *P*_adj_ = 0.015), water (R² = 0.436, *P*_adj_ = 0.015), and sediment (*R*² = 0.515, *P*_adj_ = 0.015); all four comparisons remained significant under Aitchison distance (Table S4). The comparison between *C. imbricata* and Malacalcyonacea, although showing a moderate effect size (*R*² = 0.294), was not significant after Bonferroni correction under either metric (Bray–Curtis *P*_adj_ = 0.15; Aitchison *P*_adj_ = 1.000). *Desmophyllum pertusum* and *S. variabilis* showed the most similar community composition among coral species under Bray–Curtis dissimilarity (*R*² = 0.103, *P*_adj_ = 0.030); however, this comparison was not significant under Aitchison distance (*R*² = 0.046, *P*_adj_ = 1.000; Table S4).

Intragroup variability also differed across hosts. For example, *C. imbricata* showed the lowest mean distance to centroid among coral hosts (0.309; Fig. S6), and pairwise PERMDISP comparisons confirmed that its dispersion was significantly lower than that of *S. variabilis* and Malacalcyonacea (*P*_adj_ = 0.015 for both), but did not differ significantly from *D. pertusum*, seawater or sediment (*P*_adj_ > 0.25 for all; Table S5E). This pattern was not fully preserved under Aitchison distance (Table S4f). Full pairwise and PERMDISP results for both metrics are reported in Table S4.

### Shared and host-enriched ASVs across coral taxa

The microbial communities associated with DSCs show clear patterns of both shared and host-specific ASVs (Fig. 3). The UpSet plot (Fig. 3a) shows 298 ASVs exclusive to *D. pertusum*, 140 ASVs exclusive to *C. imbricata*, 127 ASVs exclusive to *S. variabilis* and 24 ASVs exclusive to Malacalcyonacea. *Candidella imbricata* and *D. pertusum* shared the most ASVs (1275), followed by *C. imbricata, D. pertusum*, and *S. variabilis* (89 ASVs), *S. variabilis* and *D. pertusum* (88 ASVs), and Malacalcyonacea and *S. variabilis*, which shared only 7 ASVs. ASVs shared among all coral groups were only present when also shared with water and sediment samples (76 ASVs).

**Figure 3:**
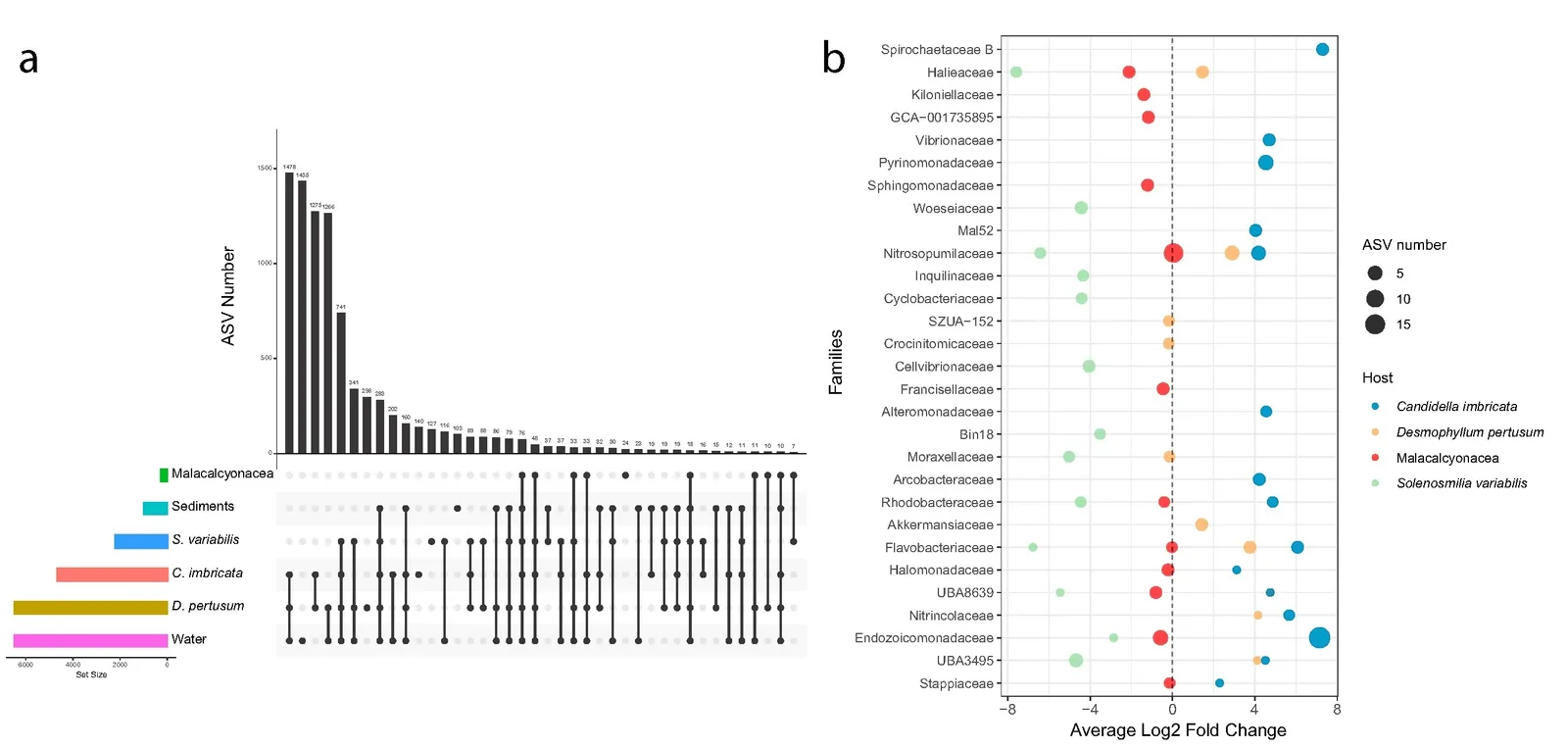
Shared and differentially abundant microbial ASVs across DSC hosts and surrounding environments. (a) UpSet plot showing the number of ASVs shared across coral species and environmental samples. Horizontal bars represent the total number of ASVs detected in each group (set size). Vertical bars represent the number of ASVs in each intersection, with dots and connecting lines below indicating which groups are involved in each intersection. (b) DESeq2 differential abundance analysis showing the average log₂ fold change per prokaryotic family across pairwise comparisons between coral species. Each point represents one family; point size indicates the number of differentially abundant ASVs in that family (adjusted *P* < 0.05, |log₂FC| > 1); colour indicates the coral host with the highest average enrichment for that family. Positive values indicate enrichment relative to other coral hosts; negative values indicate depletion.

DESeq2 analysis revealed distinct enrichment patterns for prokaryotic families across coral hosts (Fig. 3b). *Candidella imbricata* had the highest number of enriched ASVs across microbial families, with *Endozoicomonadaceae* and *Spirochaetaceae* B showing the highest average log2 fold change relative to other hosts. *Desmophyllum pertusum* had ASVs from six different families enriched, with high enrichment of *Halieaceae* and moderate enrichment of *Flavobacteriaceae*. Malacalcyonacea showed enrichment of *Inquilinaceae, Nitrosopumilaceae*, and *Endozoicomonadaceae*, among others. *S. variabilis* presented a predominantly depleted profile relative to the other coral hosts, with *Halieaceae, Flavobacteriaceae*, and *Nitrosopumilaceae* showing the lowest average log2 fold change values.

To identify prokaryotic families selectively associated with coral holobionts rather than reflecting the surrounding environmental microbial pool, we compared the relative abundance of each family in each coral species against both surrounding seawater and sediment samples (Fig. 4). When comparing coral hosts vs. seawater (Fig. 4a), *Endozoicomonadaceae* was highly enriched in *C. imbricata* (mean relative abundance ∼60%–70%), alongside *Spirochaetaceae* B, *Pyrinomonadaceae* and *Parachlamydiaceae. Desmophyllum pertusum* showed the strongest enrichment of *Halieaceae*, as well as *Crocinitomicaceae, Hyphomonadaceae, Aeribacillaceae*, and DSM-27016. *Solenosmilia variabilis* had *Halomonadaceae* as the most abundant enriched family (∼20% relative abundance), with *Inquilinaceae, Aeribacillaceae*, and *Bacillaceae* C also enriched. Malacalcyonacea showed enrichment of *Endozoicomonadaceae, Flavobacteriaceae, Bacillaceae* C, *Pyrinomonadaceae*, and *Haloarculaceae*. Notably, *Endozoicomonadaceae* was enriched in all coral hosts relative to seawater. The enrichment pattern when comparing coral hosts with sediment samples was largely consistent with the seawater comparison (Fig. 4b), with *Endozoicomonadaceae* and *Halieaceae* maintaining their host-specific enrichment in *C. imbricata* and *D. pertusum*, respectively. Additional families enriched relative to sediment included *Vibrionaceae* in *C. imbricata, Staphylococcaceae* and RKRQ01 in *D. pertusum, Anoxybacillaceae* and *Halomonadaceae* in *S. variabilis*, and *Francisellaceae, Thioglobaceae*, and *Haloarculaceae* in Malacalcyonacea. Families consistently enriched in corals relative to both environmental references, particularly *Endozoicomonadaceae* across all hosts and *Halieaceae* in *D. pertusum*.

**Figure 4:**
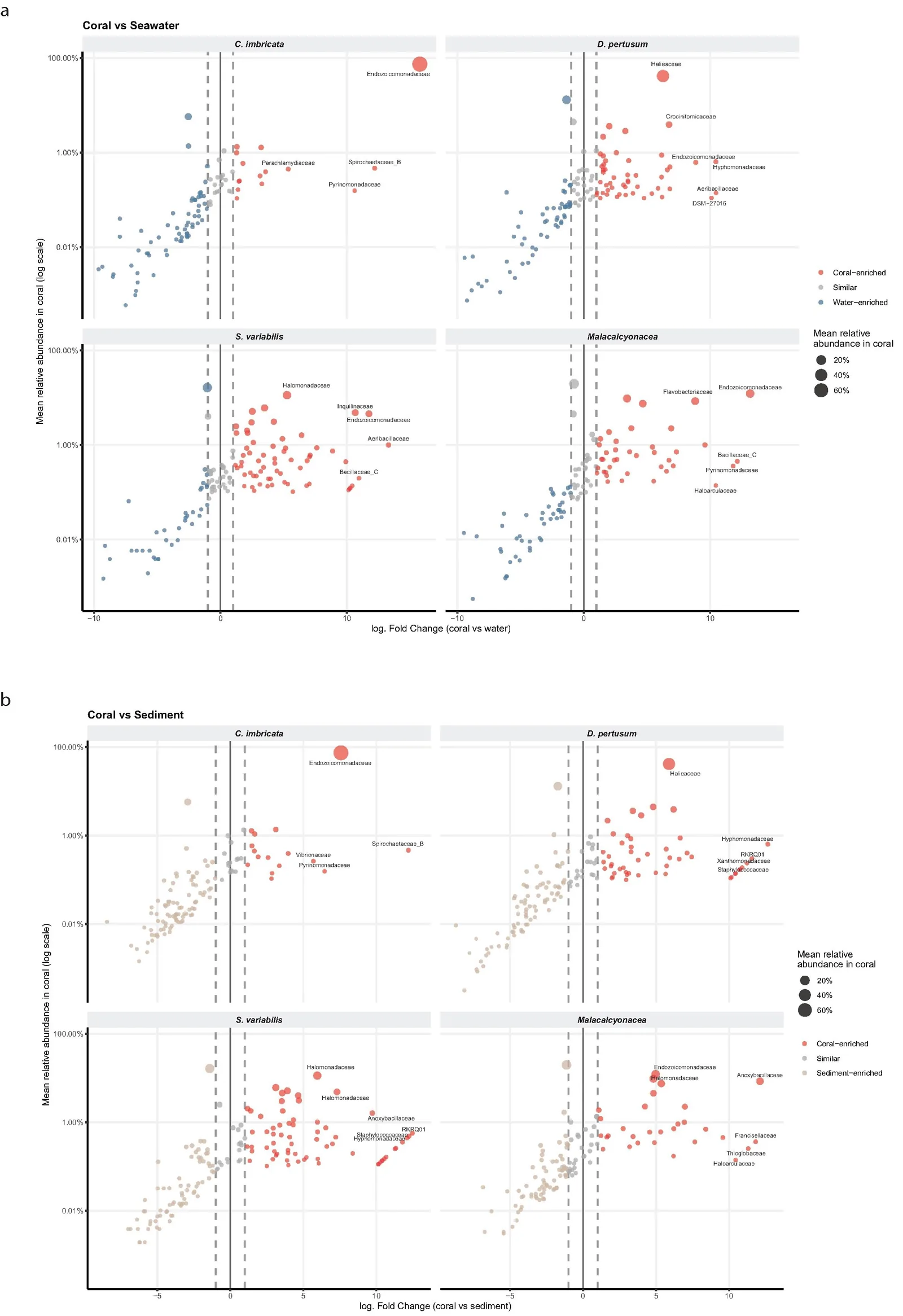
Selective enrichment of bacterial families in DSC holobionts relative to surrounding seawater and sediment. (a) Log₂ fold change (log₂FC) of mean relative abundance of each bacterial family in each coral species compared to the surrounding seawater. (b) Log₂ fold change of mean relative abundance compared to sediment. In both panels, each point represents one bacterial family; point size reflects mean relative abundance in the coral host (log scale); colour indicates enrichment category: coral-enriched (log₂FC > 1, red), similar (|log₂FC| ≤ 1, grey), or environment-enriched (log₂FC < −1, blue in panel a; beige in panel b). Dashed vertical lines indicate the |log₂FC| = 1 threshold. Labelled families are those with the highest log₂FC values per coral host. Panels are faceted by coral species.

The indicator species analysis (IndVal.g metric, 999 permutations) identified ASVs significantly associated with specific coral hosts or environmental groups (*P* ≤ 0.05). The number of indicator ASVs varied markedly across coral species: Malacalcyonacea harboured the highest number of exclusive indicators (*n* = 160), followed by *C. imbricata* (*n* = 39) and *S. variabilis* (*n* = 4). Notably, *D. pertusum* yielded no exclusive indicator ASVs (*n* = 0). The strongest indicator for *C. imbricata* was Zotu1 (IndVal = 0.859, *P* = 0.001; *Endozoicomonadaceae*), alongside Zotu19 (IndVal = 0.751, *P* = 0.009; *Endozoicomonadaceae*) and Zotu408 (IndVal = 0.735, *P* = 0.005, *Nitrincolaceae*). For *S. variabilis*, all four indicators showed moderate IndVal values (0.516–0.522, *P* ≤ 0.05). The complete list of indicator ASVs and their taxonomic assignments is provided in Table S1.

## Discussion

This study provides a comprehensive characterization of the microbiomes of six DSC species (spanning both Hexacorallia and Octocorallia) from the Campos Basin, South Atlantic Ocean, and, to our knowledge, the first to include environmental controls (seawater and sediment) for contextualizing holobiont-associated communities in this region.

Each coral species harbours a distinct microbiome that differs significantly from the surrounding seawater and sediment, and shows host-associated community differentiation. This pattern of host-associated microbial community structure is consistent with findings from other ocean basins (Kellogg *et al*. 2016, Kellogg 2019) and more recently reported for deep-water scleractinian corals from the Southwestern Atlantic (Zanotti *et al*. 2024). Among the coral species sampled, *C. imbricata* stood out as themost compositionally distinct host, with the lowest Shannon diversity (median Shannon = 1.33), the greatest separation from other groups in beta-diversity ordination, the highest intragroup homogeneity based on Bray–Curtis dissimilarity (mean distance to centroid = 0.309), with pairwise PERMDISP comparisons confirming significantly lower dispersion than *S. variabilis* and Malacalcyonacea (Bonferroni-corrected *P* = 0.015 for both; Table S4e), and the largest number of exclusive indicator ASVs (*n* = 39). Together this suggests that *C. imbricata* maintains a specialized and comparatively homogeneous microbiome, which could be consistent with a potential host-associated filtering or selection.

The dominance of *Endozoicomonadaceae* in *C. imbricata* (mean 74.4% ± 29%) likely underpins the high host specificity, low diversity (Shannon), and intragroup homogeneity. Members of the family *Endozoicomonadaceae* have been globally found to be associated with marine invertebrates, and their functional role has been postulated to span a mutualism-parasitism continuum rather than representing uniformly beneficial symbionts (da Silva *et al*. 2025). Genomic diversity within the genus *Endozoicomonas*, including divergent gene repertoires between phylogenetic clusters and evidence of pathogenic strains isolated from other marine hosts, further underscores this functional heterogeneity (Katharios *et al*. 2015, Modolon *et al*. 2025, 2026), suggesting that the ecological role of this family in *C. imbricata* cannot be assumed a priori to be beneficial. The genomic repertoire of *Endozoicomonadaceae* further suggests broad functional versatility, including pathways for carbon, nitrogen, and sulfur cycling, amino acid and B-vitamin biosynthesis, and iron acquisition via siderophores—all of which may support a mutualistic role in nutrient-limited environments such as the deep sea (Neave *et al*. 2017, da Silva *et al*. 2025). Particularly relevant in the context of the deep-sea environment is the potential role of *Endozoicomonadaceae* in dissimilatory nitrate reduction to ammonium (DNRA), which may provision ammonia to co-occurring ammonia-oxidizing archaea (*Nitrosopumilaceae*) and to the coral host under nitrogen-limiting conditions. For example, a recent study identified a novel *Endozoicomonas promiscua* sp. nov. at the same collection site as in the current study, whose genome possesses the complete DNRA pathway, supporting this hypothesis (Modolon *et al*. 2025). While Zanotti *et al*. (2024) found *Endozoicomonadaceae* to be highly variable in abundance across *D. pertusum* and *S. variabilis* from three Southwestern Atlantic basins, we observed that in *C. imbricata*, an octocoral not included in that study, was predominantly associated with this family. This suggests that the degree of association between *Endozoicomonadaceae* and DSCs may be strongly host-dependent, with some species maintaining a tight and stable symbiosis while others associate with this family only facultatively.

In contrast to *C. imbricata, D. pertusum*, one of the most widely distributed deep-sea scleractinian species in the Southwestern Atlantic (Kellogg and Pratte 2021, Carvalho *et al*. 2023), displayed a more variable and environmentally overlapping microbiome, as evidenced by the lack of exclusive indicator ASVs, intermediate intragroup dispersion, and the large number of ASVs shared with water and sediment samples. This pattern is consistent with *D. pertusum* acquiring the microbial signature of its local environment when transplanted between sites (Chapron *et al*. 2020). Furthermore, Kellogg (2019) proposed a tripartite model for the *D. pertusum* microbiome consisting of a small conserved core, a regionally consistent component, and a variable fraction shaped by local environmental conditions. Nonetheless, Zanotti *et al*. (2024) reported that *D. pertusum* exhibited the greatest stability of microbiome composition across three Southwestern Atlantic basins, indicating a comparatively lower responsiveness to environmental variation. This might be due to methodological differences in the hypervariable 16S rRNA region targeted for amplification (i.e. V4–V5 in Kellogg 2019, V1–V3 in Chapron *et al*. 2020, and V3–V4 in Zanotti *et al*. 2024), which may bias the description of community composition and thus the degree of plasticity reported. Our data support the interpretation proposed by Kellogg, as we observed selective enrichment of *Halieaceae* in *D. pertusum* relative to the surrounding environment (Fig. 4), suggesting the existence of a host-associated component, while the absence of exclusive indicator ASVs and elevated beta dispersion reflect a substantial environmentally responsive fraction of the microbiome. Nevertheless, as our sampling was restricted to a single geographic location within the Campos Basin, broader geographic inferences regarding the extent of microbiome plasticity in this species across the South Atlantic remain limited.

*Solenosmilia variabilis* and the grouped Malacalcyonacea (comprising *T. rudis, P. macrospina*, and *M. hirta*) occupied intermediate positions in the host-association continuum observed in our dataset. *Solenosmilia variabilis* yielded four exclusive indicator ASVs (IndVal = 0.516–0.522, *P* ≤ 0.05), suggesting the existence of a host-associated microbial component, albeit weak relative to *C. imbricata*. The comparatively low number of exclusive indicator ASVs, together with the fact that most differentially abundant ASVs identified for *S. variabilis* were depleted rather than enriched relative to other corals (i.e. predominantly negative log2 fold-change; Fig. 3b), may reflect a more opportunistic or generalist microbiome assembly strategy for this species. This pattern contrasts with its ecological role as a major framework-building species across the Southwestern Atlantic (Carvalho *et al*. 2023). For Malacalcyonacea, the large number of exclusive indicator ASVs (*n* = 160) must be interpreted with caution: rather than reflecting a coherent host-specific signal, it likely results from the pooling of three phylogenetically distinct octocoral species (*T. rudis, P. macrospina*, and *M. hirta*), each potentially harbouring its own distinct microbial assemblage, as supported by the high intragroup beta dispersion observed for this group (mean distance to centroid = 0.611; Table S4d), although pairwise PERMDISP comparisons indicated this dispersion did not differ significantly from that of *S. variabilis* (Table S4e), the only other coral host with comparably high dispersion, suggesting that this pattern may not be fully attributable to the pooling of multiple octocoral species. However, this interpretation is further supported by the enrichment of compositionally diverse families in Malacalcyonacea, including *Endozoicomonadaceae, Inquilinaceae, Nitrosopumilaceae*, and *Haloarculaceae* (Fig. 4), which may reflect taxon-specific associations rather than a shared octocoral signal. The grouping of these species was done because of the low per-species replication (*n* = 1–2), which resulted from their cryptic morphology and the difficulty of species-level identification after collection and before molecular analysis. Future studies with targeted, replicated sampling of these octocoral taxa will be essential to disentangle species-level host specificity within this group and to assess whether the microbial patterns observed here are consistent across the South Atlantic.

The collection site in the Campos Basin is located within one of Brazil’s most active oil- and gas-producing regions, and the abiotic parameters measured at the site reflect this context, with total hydrocarbon concentrations reaching 24.12 ± 9.89 μg/l, including detectable aliphatic fractions (Table S2). While background dissolved hydrocarbon concentrations specific to deep-water settings remain poorly characterized, values of this magnitude exceed typical background concentrations reported for offshore waters (∼0.1 μg/l; Beyer *et al*. 2020) and fall within the range reported for dissolved hydrocarbon plumes at comparable depths associated with oil and gas infrastructure in other basins [up to 78 μg/l at ∼1100 m during the Deepwater Horizon blowout, which was documented to affect deep-water coral communities in the Gulf of Mexico; (Reddy *et al*. 2012, White *et al*. 2012, Valentine *et al*. 2014)]. These values are therefore consistent with measurable influence from the oil and gas exploration and production activities characteristic of the Campos Basin.

While functional activity cannot be inferred from 16S rRNA gene data alone, the selective enrichment of *Halieaceae*, a family containing known hydrocarbon-degrading members, raises the hypothesis that hydrocarbon-associated microbial metabolism may form part of the *D. pertusum*’s response to this environmental context (Peeb *et al*. 2022, Zhang *et al*. 2022), although the hydrocarbon-degrading capacity described for this family in other systems has not been confirmed in the specific coral-associated ASVs identified here. Metagenomic or functional analyses will be required to test whether these taxa actively contribute to hydrocarbon degradation or holobiont resilience. More broadly, to our knowledge, our study provides the first characterization of DSC-associated microbiomes that explicitly incorporates both octocoral and scleractinian hosts from the Campos Basin, establishing a baseline microbial dataset against which future changes can be assessed, and documenting the differential degrees of host-associated microbial differentiation. Given the ongoing pressures of oil and gas exploration and projected climate change, monitoring the microbiomes of these ecologically important reef-builders is a critical step towards evidence-based stewardship of deep-sea ecosystems.

## Supplementary Material

qvag048_Supplemental_Files

## Data Availability

The data underlying this article are available at the National Centre for Biotechnology Information (NCBI) under BioProject ID PRJNA1193098.
