# Supplementary material for: Contrasting microbiomes in deep-sea scleractinian and octocoral species from the South Atlantic Ocean
Source: Sustain Microbiol. 2026 Oct 15;3(4):qvag048. doi: 10.1093/sumbio/qvag048 (PMC13712126; doi:10.1093/sumbio/qvag048)
Supplement: Supplementary file 1 [file qvag048_supplemental_files.zip › Hill & Damasceno et al_26_SM_after review.docx]

**Supplementary Material - Deep-sea coral microbiomes in the South Atlantic**

**Figure S1.** Representative *in situ* photographs of the eight deep-sea coral species collected in the Campos Basin during the ProBioDeep expedition (2021). (A) *Desmophyllum pertusum* (n = 13), (B) *Solenosmilia variabilis* (n = 11), (C) *Candidella imbricata* (n = 7), (D) *Trachythela rudis* (n = 2), (E) *Paramuricea macrospina* (n = 2), (F) *Paramuricea biscaya* (n = 1), (G) *Muriceides* cf. *hirta* (n = 1), (H) *Thouarella viridis* (n = 1). Images were captured by ROV-mounted cameras at approximately 710 m depth. White arrowheads indicate the specimen of interest in images where more than one organism is present.

**Figure S2.** Maximum-likelihood phylogenetic tree of Octocorallia based on a concatenated alignment of cox1, igr1 and mtMutS gene regions (1,000 bootstrap replicates). Sequences from this study are highlighted in colour: *Candidella imbricata* specimens (C1–C7) in green and Malacalcyonacea specimens (M1–M5) in blue. Reference sequences retrieved from NCBI are shown in black. Bootstrap support values are indicated at internal nodes. The best-fit substitution model was selected independently for this dataset using the “Find Best DNA/Protein Models (ML)” function in MEGA prior to tree inference. Specimen C7, although not fully resolved within the *Candidella imbricata* clade, was confirmed as this species by BLAST analysis (99.9% identity to NC062002); its placement likely reflects the limited phylogenetic resolution resulting from the scarcity of reference sequences for this species in public databases.

**Figure S3.** Maximum-likelihood phylogenetic tree of *Solenosmilia variabilis* based on the mitochondrial cytochrome c oxidase subunit I (COI) gene (1,000 bootstrap replicates). Sequences from this study (S1–S11) are shown in bold. Reference sequences retrieved from NCBI are shown in regular font. Bootstrap support values are indicated at internal nodes. The best-fit substitution model was selected using the “Find Best DNA/Protein Models (ML)” function in MEGA prior to tree inference. *Paragorgia coralloides* (KF785800) was used as outgroup.

**Figure S4.** Maximum-likelihood phylogenetic tree of *Desmophyllum pertusum* based on the mitochondrial cytochrome c oxidase subunit I (COI) gene (1,000 bootstrap replicates). Sequences from this study (D1–D13) are shown in bold. Reference sequences retrieved from NCBI are shown in regular font. Bootstrap support values are indicated at internal nodes. The best-fit substitution model was selected using the “Find Best DNA/Protein Models (ML)” function in MEGA prior to tree inference. *Paragorgia coralloides* (KF785800) was used as outgroup. FR821799 *Lophelia pertusa* is a currently accepted synonym of *Desmophyllum pertusum* and is included for reference.

**Figure S5.** Host-specific enrichment of Endozoicomonadaceae across deep-sea coral species. Box plots showing the relative abundance (%) of the family Endozoicomonadaceae in each coral host group. Boxes represent the interquartile range, horizontal lines the median, whiskers extend to 1.5× the interquartile range, and individual points represent samples. Candidella imbricata shows markedly higher and more consistent enrichment of Endozoicomonadaceae relative to all other coral hosts.

**Figure S6.** Intragroup microbiome variability assessed by PERMDISP analysis. Box plots showing the distance to group centroid (Bray–Curtis dissimilarity) for each sample group, as calculated by the PERMDISP test. Lower values indicate more homogeneous microbial communities within a group. Candidella imbricata displays the lowest intragroup variability among coral hosts (mean distance to centroid = 0.309), while Malacalcyonacea shows the highest (0.611), consistent with this group comprising three phylogenetically distinct octocoral species (Trachythela rudis, Paramuricea macrospina and Muriceides hirta). PERMDISP: F = 5.664, p = 0.002.

**Table S1.** Indicator Species Analysis results (i.e., “IndicSpecies” sheet) identifying ASVs significantly associated with each coral species, based on the Indicator Value method implemented in the indicspecies R package (multipatt function; association function IndVal.g; 999 permutations; α = 0.05). For each group, A denotes specificity (the probability that a sample belongs to the target group given the presence of the ASV), B denotes fidelity (the probability of the ASV occurring in samples of the target group). Stat is the IndVal.g statistic, calculated as the square root of the product of A and B. Reported ASVs met the significance threshold (p ≤ 0.05); asterisks denote significance levels (* p ≤ 0.05, ** p ≤ 0.01, *** p ≤ 0.001). Taxonomic assignment (family, genus, species) for each ASV is provided in the “Taxonomy” sheet. Excel file attached.

**Table S2.**  Abiotic parameters measured at the coral collection sites in the Campos Basin (mean ± SD, n = 4 measurements).

| Abiotic Measurements | |
| --- | --- |
| Temperature (^o^C) | 5.31 ± 0.76 |
| Depth (m) | 710 ± 80 |
| Amonium (µmol/L) | 1.46 ± 0.84 |
| Nitrite (µmol/L) | 0.06 ± 0.03 |
| Nitrate (µmol/L) | 27.81 ± 2.62 |
| Phosphate (µmol/L) | 1.51 ± 2.03 |
| DOC (µg/L) | 3.46 ± 3.10 |
| Total HC (µg/L) | 24.12 ± 9.89 |
| C-16 to C-21 – Aliphatic (µg/L) | 5.77 ± 0.84 |
| C-21 to C-34 – Aliphatic (µg/L) | 17.53 ± 9.48 |

**Table S3.** Primer sequences and PCR conditions used for molecular identification of coral specimens. Gene regions amplified include the mitochondrial cytochrome c oxidase subunit I (COI) for scleractinian corals, and the cytochrome oxidase I–intergenic region (cox1+igr1) and mismatch repair protein gene (mtMutS) for octocorals. For each gene region, forward and reverse primer sequences, thermocycling conditions, and the corresponding literature source are indicated.

| **Gene region** | **Primer** | **Sequence (5' -> 3')** | **PCR profile** | **Reference** |
| --- | --- | --- | --- | --- |
| COI | LCO1490 | GGT CAA CAA ATC ATA AAG ATA TTG G | 95°:3 min + 30 cycles of 95°:60 s, 45°:60 s, 72°:90 s + 72°: 7 min | Soto et al. (2021) |
| COI | HCO2198 | TAA ACT TCA GGG TGA CCA AAA AAT CA |  |  |
| cox1+igr1 | COII8068F | CCA TAA CAG GAC TAG CAG CAT C | 94°:5 min + 32 cycles of 94°:30 s, 60°:90 s, 72°:60 s + 72°:10 min | Radice et al. (2016); Quattrini et al. (2014); McFadden et al. (2011); France & Hoover (2002) |
| cox1+igr1 | COIOCTR | ATC ATA GCA TAG ACC ATA CC |  |  |
| mtMutS | ND42599F | GCC ATT ATG GTT AAC TAT TAC | 94°:5 min + 32 cycles of 94°:30 s, 51°:45 s, 72°:60 s + 72°:10 min | Radice et al. (2016); McFadden et al.(2011); Sánchez et al. (2003) |
| mtMutS | MUT3458R | TSG AGC AAA AGC CAC TCC |  |  |

**Table S4.** Sensitivity of beta diversity results to the choice of dissimilarity metric (Bray-Curtis vs. Aitchison distance). Beta diversity analyses were repeated using Aitchison distance (Euclidean distance on centred log-ratio-transformed abundances, following Bayesian-multiplicative zero replacement; cmultRepl, method CZM, zCompositions package) to assess robustness to metric choice, following the approach of Fuschi et al. (2025). ASVs present in fewer than three samples were excluded prior to zero replacement for the Aitchison analysis; the full ASV set was retained for Bray-Curtis. Pairwise p-values were Bonferroni-corrected across all 15 pairwise comparisons within each metric.

A. Global PERMANOVA

| **Metric** | **R²** | **F** | **p** |
| --- | --- | --- | --- |
| Bray-Curtis | 0.399 | 6.771 | < 0.001 |
| Aitchison | 0.371 | 6.021 | 0.001 |

B. Pairwise PERMANOVA — Both metrics (Bonferroni-adjusted)

| **Pairwise comparison** | **Bray-Curtis R²** | **Bray-Curtis p_adj** | **Aitchison R²** | **Aitchison p_adj** |
| --- | --- | --- | --- | --- |
| *Water vs Malacalcyonacea* | 0.227 | 0.030 | 0.180 | 0.015 |
| *Water vs D. pertusum* | 0.324 | 0.015 | 0.255 | 0.015 |
| *Water vs S. variabilis* | 0.261 | 0.015 | 0.273 | 0.015 |
| *Water vs C. imbricata* | 0.436 | 0.015 | 0.212 | 0.015 |
| *Water vs Sediment* | 0.376 | 0.015 | 0.318 | 0.015 |
| *Malacalcyonacea vs D. pertusum* | 0.160 | 0.015 | 0.137 | 0.180 (n.s.) |
| *Malacalcyonacea vs S. variabilis* | 0.115 | 0.015 | 0.201 | 0.015 |
| *Malacalcyonacea vs C. imbricata* | 0.294 | 0.165 (n.s.) | 0.098 | 1.000 (n.s.) |
| *Malacalcyonacea vs Sediment* | 0.238 | 0.015 | 0.214 | 0.075 (n.s.) |
| *D. pertusum vs S. variabilis* | 0.103 | 0.015 | 0.046 | 1.000 (n.s.) |
| *D. pertusum vs C. imbricata* | 0.332 | 0.015 | 0.151 | 0.015 |
| *D. pertusum vs Sediment* | 0.331 | 0.015 | 0.358 | 0.015 |
| *S. variabilis vs C. imbricata* | 0.268 | 0.015 | 0.224 | 0.015 |
| *S. variabilis vs Sediment* | 0.276 | 0.015 | 0.406 | 0.015 |
| *C. imbricata vs Sediment* | 0.515 | 0.015 | 0.282 | 0.015 |

C. PERMDISP - Global test, both metrics

| **Metric** | **F** | **df** | **p** |
| --- | --- | --- | --- |
| Bray-Curtis | 5.664 | 5.51 | 0.001 |
| Aitchison | 4.010 | 5.51 | 0.004 |

D. PERMDISP - Mean distance to centroid by group, both metrics

| **Group** | **Bray-Curtis** | **Aitchison** |
| --- | --- | --- |
| *C. imbricata* | 0.309 | 48.95 |
| *D. pertusum* | 0.527 | 47.00 |
| *S. variabilis* | 0.609 | 41.57 |
| *Malacalcyonacea* | 0.611 | 60.58 |
| *Water* | 0.418 | 77.85 |
| *Sediment* | 0.366 | 78.14 |

E. PERMDISP – pairwise comparisons, Bray-Curtis dissimilarity

| **Group 1** | **Group 2** | **F** | **df** | **p** | **p (Bonferroni)** |
| --- | --- | --- | --- | --- | --- |
| *Desmophyllum pertusum* | *Solenosmilia variabilis* | 3.840 | 1, 22 | 0.071 | 1.000 (n.s.) |
| *Desmophyllum pertusum* | *Candidella imbricata* | 6.221 | 1, 18 | 0.011 | 0.165 (n.s.) |
| *Desmophyllum pertusum* | Malacalcyonacea | 2.031 | 1, 16 | 0.190 | 1.000 (n.s.) |
| *Desmophyllum pertusum* | Water | 3.292 | 1, 24 | 0.099 | 1.000 (n.s.) |
| *Desmophyllum pertusum* | Sediment | 6.795 | 1, 19 | 0.011 | 0.165 (n.s.) |
| *Solenosmilia variabilis* | *Candidella imbricata* | 13.382 | 1, 16 | 0.001 | 0.015 |
| *Solenosmilia variabilis* | Malacalcyonacea | 0.012 | 1, 14 | 0.913 | 1.000 (n.s.) |
| *Solenosmilia variabilis* | Water | 12.191 | 1, 22 | 0.001 | 0.015 |
| *Solenosmilia variabilis* | Sediment | 24.544 | 1, 17 | 0.001 | 0.015 |
| *Candidella imbricata* | Malacalcyonacea | 6.001 | 1, 10 | 0.001 | 0.015 |
| *Candidella imbricata* | Water | 1.223 | 1, 18 | 0.323 | 1.000 (n.s.) |
| *Candidella imbricata* | Sediment | 0.259 | 1, 13 | 0.612 | 1.000 (n.s.) |
| Malacalcyonacea | Water | 5.908 | 1, 16 | 0.034 | 0.510 (n.s.) |
| Malacalcyonacea | Sediment | 12.419 | 1, 11 | 0.001 | 0.015 |
| Water | Sediment | 0.496 | 1, 19 | 0.514 | 1.000 (n.s.) |

F. PERMDISP – pairwise comparisons, Aitchison distance

| **Group 1** | **Group 2** | **F** | **df** | **p** | **p (Bonferroni)** |
| --- | --- | --- | --- | --- | --- |
| *Desmophyllum pertusum* | *Solenosmilia variabilis* | 0.627 | 1, 22 | 0.433 | 1.000 |
| *Desmophyllum pertusum* | *Candidella imbricata* | 0.463 | 1, 18 | 0.496 | 1.000 |
| *Desmophyllum pertusum* | Malacalcyonacea | 0.631 | 1, 16 | 0.447 | 1.000 |
| *Desmophyllum pertusum* | Water | 5.460 | 1, 24 | 0.028 | 0.420 |
| *Desmophyllum pertusum* | Sediment | 7.777 | 1, 19 | 0.012 | 0.180 |
| *Solenosmilia variabilis* | *Candidella imbricata* | <0.001 | 1, 16 | 0.989 | 1.000 |
| *Solenosmilia variabilis* | Malacalcyonacea | 1.885 | 1, 14 | 0.197 | 1.000 |
| *Solenosmilia variabilis* | Water | 9.678 | 1, 22 | 0.006 | 0.090 |
| *Solenosmilia variabilis* | Sediment | 14.496 | 1, 17 | 0.001 | 0.015 |
| *Candidella imbricata* | Malacalcyonacea | 1.527 | 1, 10 | 0.250 | 1.000 |
| *Candidella imbricata* | Water | 7.366 | 1, 18 | 0.017 | 0.255 |
| *Candidella imbricata* | Sediment | 13.871 | 1, 13 | 0.004 | 0.060 |
| Malacalcyonacea | Water | 0.675 | 1, 16 | 0.419 | 1.000 |
| Malacalcyonacea | Sediment | 1.497 | 1, 11 | 0.229 | 1.000 |
| Water | Sediment | 0.186 | 1, 19 | 0.645 | 1.000 |

**Table S5.** Sequencing depth per sample at each processing step, and alpha diversity calculated on rarefied data. Excel file attached.
